# Span 60/Cholesterol Niosomal Formulation as a Suitable Vehicle for Gallic Acid Delivery with Potent In Vitro Antibacterial, Antimelanoma, and Anti-Tyrosinase Activity

**DOI:** 10.3390/ph16121680

**Published:** 2023-12-02

**Authors:** Sara Zolghadri, Ali Ghanbari Asad, Fatemeh Farzi, Fatemeh Ghajarzadeh, Zeinab Habibi, Mahdie Rahban, Samaneh Zolghadri, Agata Stanek

**Affiliations:** 1 Department of Chemistry, Jahrom Branch, Islamic Azad University, Jahrom 7414785318, Iran; sarazj1371@gmail.com; 2 Department of Medical Biotechnology, Fasa University of Medical Sciences, Fasa 7461686688, Iran; alighanbariasad@gmail.com; 3 Department of Biology, Jahrom Branch, Islamic Azad University, Jahrom 7414785318, Iran; fatemehfrz09@gmail.com (F.F.); fatemehghajarzadeh1372@gmail.com (F.G.); zynb00220@gmail.com (Z.H.); 4 Neuroscience Research Center, Institute of Neuropharmacology, Kerman University of Medical Sciences, Kerman 7616913555, Iran; mrohban@ut.ac.ir; 5 Department and Clinic of Internal Medicine, Angiology and Physical Medicine, Faculty of Medical Sciences in Zabrze, Medical University of Silesia, Batorego 15 St, 41-902 Bytom, Poland

**Keywords:** entrapment efficiency, gallic acid niosomes, Span 60, drug delivery, melanoma, tyrosinase inhibitor

## Abstract

Natural compounds such as gallic acid (GA) have attracted more attention in cosmetic and pharmaceutical skin care products. However, the low solubility and poor stability of GA have limited its application. This study aimed to synthesize and characterize the GA niosomal dispersion (GAN) and investigate the potential of an optimal formulation as a skin drug delivery system for GA. For this purpose, GAN formulations were synthesized using the thin layer evaporation method with different molar ratios of Tween 60/Span 60, along with a constant molar ratio of polyethylene glycol 4000 (PEG-4000) and cholesterol in a methanol and chloroform solvent (1:4 *v*/*v*). The physicochemical properties of nanosystems in terms of size, zeta potential, drug entrapment, drug release, morphology, and system–drug interaction were characterized using different methods. In addition, in vitro cytotoxicity, anti-tyrosinase activity, and antibacterial activity were evaluated by MTT assay, the spectrophotometric method, and micro-well dilution assay. All formulations revealed a size of 80–276 nm, polydispersity index (PDI) values below 0.35, and zeta potential values below—9.7 mV. F2 was selected as the optimal formulation due to its smaller size and high stability. The optimal formulation of GAN (F2) was as follows: a 1:1 molar ratio of Span 60 to cholesterol and 1.5 mM GA. The release of the F2 drug showed a biphasic pattern, which was fast in the first 12 h until 58% was released. Our results showed the high antibacterial activity of GAN against *Escherichia coli* and *Pseudomonas aeruginosa*. The MTT assay showed that GA encapsulation increased its effect on B6F10 cancer cells. The F2 formulation exhibited potent anti-tyrosinase activity and inhibited melanin synthesis. These findings suggest that it can be used in dermatological skin care products in the cosmetic and pharmaceutical industries due to its significant antibacterial, anti-melanoma, and anti-tyrosinase activity.

## 1. Introduction

Gallic acid (GA), also known as 3,4,5-trihydroxybenzoic acid, is a natural phenolic molecule with different therapeutic properties [1], including antibacterial [2,3], anti-inflammatory [4], anti-obesity, and anticancer activity [5,6]. Furthermore, GA and its derivatives have shown potent tyrosinase (TYR) inhibitory activity [2]. TYR is a multi-copper enzyme that plays an important role in melanogenesis and enzymatic browning. Melanin is a natural pigment that protects the skin against harmful ultraviolet irradiation (UVR; ~320 to 400 nm). However, excess melanin production induces significant pathological conditions, such as hyperpigmentation, melasma, and melanoma [7]. To prevent melanin overproduction and accumulation in the skin, several TYR inhibitors have been identified to date [8,9]. In addition, GA has exhibited antibacterial activity against various bacteria, including *Escherichia coli*, *Helicobacter pylori*, *Streptococcus mutans*, *Staphylococcus aureus*, *Listeria monocytogenes*, and *Pseudomonas aeruginosa* [10,11]. In phenolic compounds, the location and quantity of the hydroxyl groups on the phenol group are linked to their toxicity toward microorganisms. Studies have indicated that more hydroxylation is associated with higher toxicity levels [10,12,13]. Numerous studies have shown that GA with four hydroxyl groups is effective as an antibacterial agent by altering membrane structure and bacterial metabolism and inhibiting biofilm formation [14]. Thus, natural compounds such as GA with TYR inhibitory activity, antimicrobial potential, and antioxidant effects have attracted more attention in cosmetic and pharmaceutical products.

Despite the beneficial properties of GA, its low solubility and susceptibility to environmental factors have limited its application in the medicinal and pharmaceutical industries. GA is prone to oxidation when exposed to oxygen, leading to degradation and the loss of its advantageous properties. Similarly, exposure to light and extreme temperatures can cause the degradation of GA and reduce its effectiveness [15,16,17]. In addition, the rapid metabolism of GA reduces its half-life and limits its bioavailability and systemic exposure after administration [18,19]. Therefore, these properties of GA pose challenges in achieving sustained therapeutic levels of the compound in pharmaceutical approaches. To overcome these limitations, several procedures, including drug delivery systems such as niosomes [12,15,20,21,22], have been proposed to encapsulate GA, protecting it from degradation and facilitating its targeted delivery to specific sites in the body [23,24,25]. 

Niosomes, composed of non-ionic surfactants, have gained significant attention in pharmaceutical research [26,27]. Non-ionic surfactants can form stable niosomes due to their long hydrocarbon chains without double bonds. They have desirable and attractive features like biodegradability, biocompatibility, and low toxicity. Tween, Span, and Brij are known non-ionic surfactants in the preparation of niosomes. Span is often used in combination with one or more of the Tween products to achieve a desired high hydrophilic–lipophilic balance (HLB) value. However, they are unable to form niosomes without appropriate stabilizers and additives [28]. Cholesterol is the most common additive that influences the packing arrangement of surfactant molecules and lipids, affecting the size and morphology. Typically, the incorporation of cholesterol enhances the rigidity of the bilayer and reduces fluidity, thereby improving stability and reducing aggregation tendencies [29]. The specific impact of cholesterol and surfactants on nanoniosome size can vary depending on molar ratios, preparation methods, and overall composition [30]. The particle size of a drug delivery system plays a crucial role in its performance, affecting stability, solubility, and bioavailability properties [31]. A narrow size distribution is desirable for effective delivery. Reducing particle size can lead to improved solubility and improved bioavailability of encapsulated substances, highlighting the importance of size control in colloidal carriers for optimizing drug delivery efficacy [32]. Niosomes are very similar to liposomes but with a different lipid composition; they self-assemble into closed vesicles capable of encapsulating hydrophilic and lipophilic drugs [33,34]. These vesicular drug delivery systems are in the form of small unilamellar, multilamellar, or large unilamellar vesicles with numerous advantages, including improved drug stability, enhanced solubility, controlled release, and the potential for targeted delivery to specific tissues or cells [35,36]. It is also necessary to know the behavior of the particles in the medium to be transported. This characteristic is given by the charge of the particles, as assessed by the zeta potential. The zeta potential plays a crucial role in niosome stability and their behavior in different environments. Niosomes with low zeta potential values are more susceptible to aggregation and potential destabilization. If the zeta potential is near zero, the particles will aggregate because they have a low electrostatic repulsion. Generally, when the zeta potential is greater than 35 mV or lower than −35 mV, the particles are considered stable [37]. 

Compared with other carriers, niosomes are attractive carriers for topical administration of active agents because they have high stability and cause less irritation during their action in the dermal route [38,39]. They allow drug delivery into the skin by increasing the residence time of drugs in the stratum corneum and epidermis. Furthermore, it appears that niosomes can protect drugs against the effects of bacterial enzymes. Niosomes interact with bacterial cell membranes through fusion and contact release mechanisms, facilitating the direct delivery of their encapsulated drugs onto or inside bacterial cells for targeted action [40]. Recently, many researchers have investigated drug-loaded niosomes to find more effective and selective and less toxic alternative therapeutics for skin disorders, such as hyperpigmentation [41], melasma [42], melanoma [43,44,45], acne, and other problems [46]. However, particle size plays a vital role in drug delivery through the stratum corneum, and large particles are unable to diffuse through the skin layers and reach the target tissue. For example, particles with a size of 50 nm display better diffusion than those of 200 nm [47]. Further research is needed to understand the relationship between skin permeability and particle size. 

This study aimed to synthesize and characterize three niosomal formulations, including Tween 60/cholesterol, Span 60/cholesterol, and Span 60/Tween 60/cholesterol, and find an appropriate skin drug delivery system for GA, especially in terms of size and stability. In addition, the cytotoxicity of the optimal formulation of GAN against B16F10 melanoma cells and the antibacterial and TYR inhibitory activity were investigated compared with those of pure GA for cosmetic and pharmaceutical applications.

## 2. Results 

### 2.1. Niosomal Formulations Obtained and Their Physicochemical Characteristics 

Niosomal dispersions were prepared in three formulations (F1, F2, and F3) using the thin film hydration method and employing a mixture of amphiphilic surfactants (Span^TM^ 60 and Tween^®^ 60) at different molar ratios and cholesterol. The total surfactant-to-cholesterol ratio was fixed to examine the effect of the surfactant type on the properties of the niosomal dispersions. Table 1 presents the surfactant-to-cholesterol ratios and the GA concentration of the formulations. The results revealed variations in size, zeta potential, and EE% between the GAN formulations with different surfactant types. Also, there were positive correlations between size and EE% (R = 0.97), size and zeta potential (R = 0.98), and EE% and zeta potential (R = 0.92). The PDI values obtained ranged from 0.22 (F1) to 0.34 (F3). In addition, the F2 formulation showed the lowest particle size. 

On the basis of these results, the F2 formulation (cholesterol—span 60 1:1, molar ratio) was chosen for further investigation due to its smaller size and lower zeta potential, indicating better stability and the potential for enhanced transdermal drug delivery despite the lower EE%.

### 2.2. Morphological Characterization of GAN with F2 Formulation

**Figure 1:**
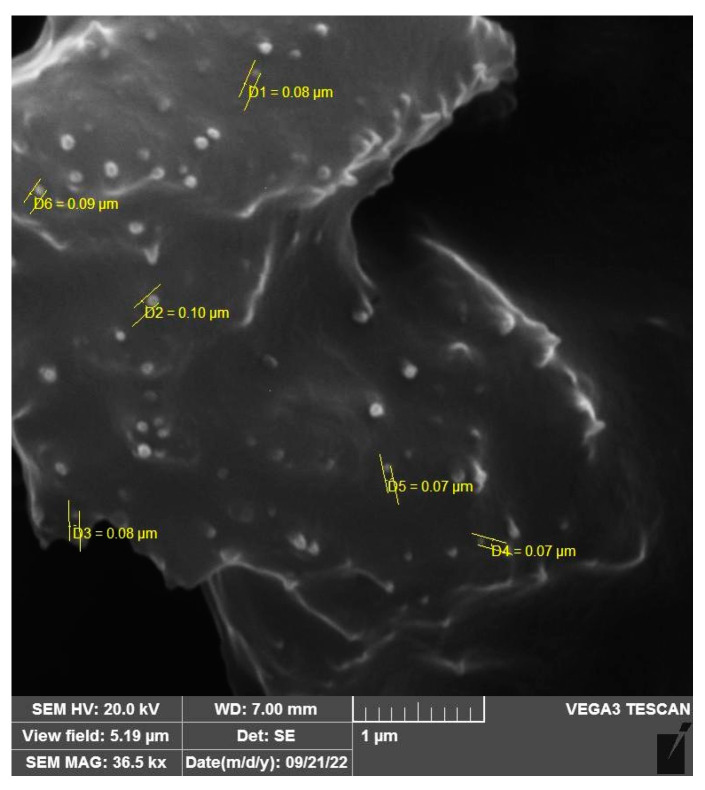
Morphological characterization of optimal GAN formulation (F2) by scanning electron microscopy.

The morphology of the optimal GAN formulation (F2) was examined using scanning electron microscopy, which confirmed a consistent spherical shape, a smooth surface, and an average size of 65 nm for the GAN without aggregation (Figure 1). 

### 2.3. FTIR Analysis of F2 Formulation 

**Figure 2:**
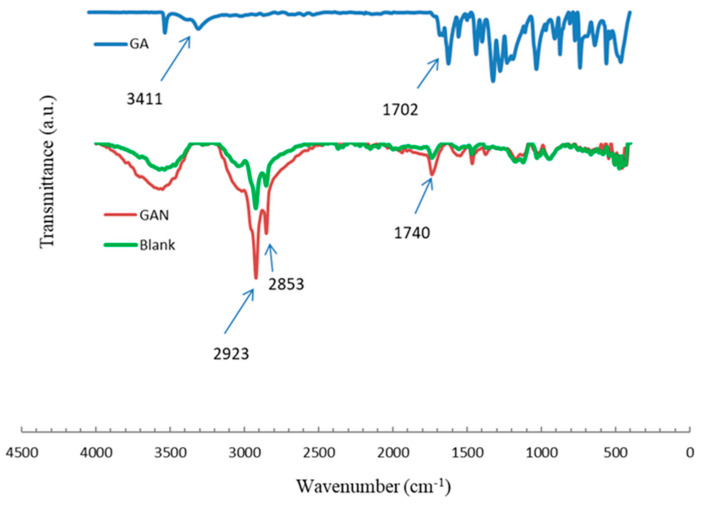
FTIR spectra of GA, blank nanoniosome (F02), and GAN (F2).

As highlighted in Figure 2, GA demonstrates different spectra from GAN (F2 formulation) and the blank niosomal formulation (Figure 2). The FTIR spectrum of GA displays distinct peaks and bands that offer valuable information on its molecular structure. The sharp and broad band between 3600 and 2500 cm^−1^ and the narrow peak at 1702 cm^−1^ indicate the stretching vibrations of the OH and carbonyl group, respectively, confirming the presence of a carboxyl group in GA. Additionally, three peaks observed at 1450, 1541, and 1616 cm^−1^ correspond to the stretching vibrations of C–C bonds in an aromatic ring, a characteristic feature of GA’s structure. Furthermore, several peaks between 1300–1000 cm^−1^ can be attributed to the stretching vibrations of C–O bonds and the bending vibration of O–H bonds in GA. A significant finding was the disappearance of the prominent characteristic peaks of GA in the final optimal niosomal formulation of F2. 

### 2.4. GA Release 

**Figure 3:**
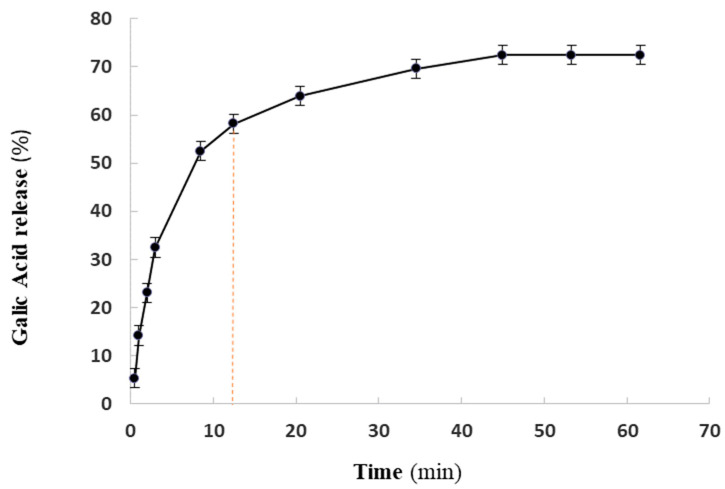
In vitro release profile of GA from GAN (F2) at 37 °C and pH 7.2. The first stage of release (0 up to 58%) is shown by dash line.

Drug release is a critical factor in drug delivery systems. The cumulative amounts of GA released from the F2 formulation in 62 h (Figure 3) showed a biphasic profile (two stages) for the release of GA from the nanoniosomal structures. The results showed a biphasic profile (two stages) for the release of GA from the F2 formulation. To understand the release kinetics of the drug delivery system, linear plots for the first stage of release (0 up to 58%) were constructed using different kinetics models, including the Korsmeyer–Peppas, Higuchi, first-order, and zero-order models, and the regression coefficient of the linear curve was calculated (Table 2).

### 2.5. Inhibitory and Bactericidal Concentration 

The MIC and MBC values of GA, GAN (F2 formulation), and gentamycin against *E. coli*, P. aeruginosa, and K. pneumonia are presented in Table 3. According to the results, the F2 formulation exhibited potent antimicrobial activity against *E. coli* (with an MIC of 23 µM and an MBC of 46 µM) and *P. aeruginosa* (with an MIC of 187 µM and an MBC of 375 µM) compared with GA and gentamycin. 

### 2.6. Cytotoxicity Effect of F2 Formulation

The viability of the *B16F10* cell line was assessed using the MTT assay to evaluate the effect of the F2 formulation. The MTT assay results provided crucial evidence supporting the capability of the niosomal dispersions to deliver the drug effectively, promoting its cytotoxic effects on cancer cells. 

**Figure 4:**
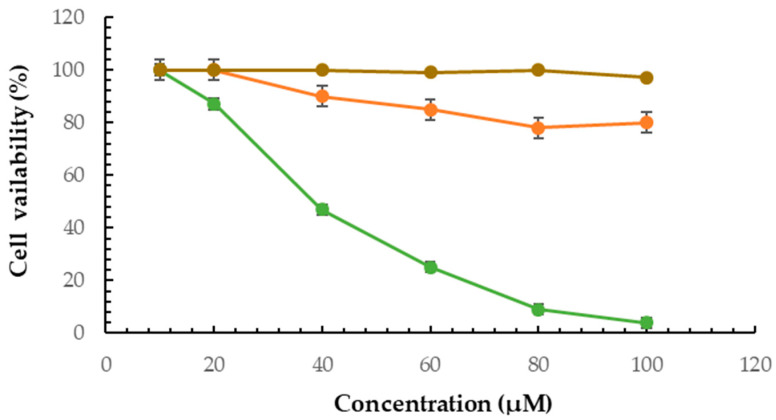
Cytotoxicity assay of GA (orange), blank niosomal dispersion (brown), and GAN (green) on *B16F10* cells.

The results showed that GA had no significant cytotoxicity effect against *B16F10* cells. However, GAN induced a potent cytotoxicity effect against cells (*p* < 0.05) (Figure 4).

### 2.7. Melanin Assay

**Figure 5:**
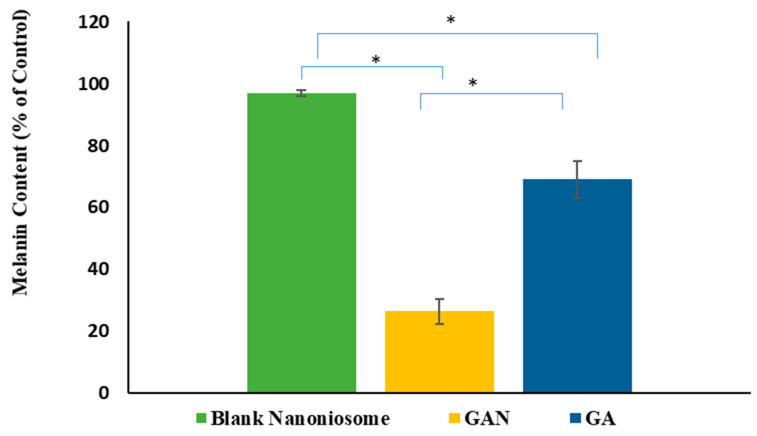
The melanin content of B16F10 melanoma cells treated with blank niosomal F2 dispersion, GA, and GAN (* *p* < 0.05).

The melanin content of cells treated with GA and GAN was assessed by ELISA. The results showed higher inhibitory activity of GAN (*p* < 0.05) on melanin synthesis compared with GA (Figure 5).

### 2.8. Intracellular TYR Activity

**Figure 6:**
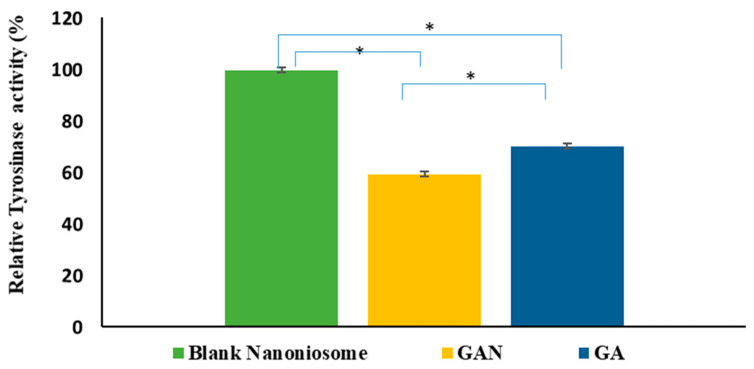
The percentage of tyrosinase activity of B16F10 melanoma cells treated with blank niosomal F2 dispersion, GA, and GAN (* *p* < 0.05).

In this study, intracellular TYR activity was measured to assess the inhibitive effect of GA and GAN on melanogenesis. The results indicated that GA and GAN could significantly (*p* < 0.05) inhibit cellular TYR activity, but GAN had more of an inhibitory effect on the cellular TYR activity (Figure 6).

## 3. Discussion

In this study, niosomal formulations (F1, F2, and F3) were synthesized and characterized as GA delivery systems. The PDI values ranged from 0.22 (F1) to 0.34 (F3), indicating a uniform size distribution. In addition, the F2 formulation showed the lowest particle size. According to the zeta potential results, all the GAN formulations had a negative charge, indicating their potential for stable behavior and a reduced tendency for aggregation. As shown in Table 1, at a fixed amount of cholesterol, the highest EE (96 ± 1%) was observed for F3 (1:1 molar ratio of cholesterol to Tween 60), and the lowest EE (75 ± 2%) was recorded for F2 (1:1 molar ratio of Span 60 to cholesterol). This means that at the same concentration of wall material, EE decreased by increasing the amount of Span 60 in the surfactant ratio. Also, a direct proportional relationship between EE and the vesicle particle size was observed, consistent with previous findings that encapsulation efficiencies are mainly affected by the content and type of the surfactants [48,49]. The EE value provides valuable information on the percentage of the drug entrapped in the nanoniosomes. It is essential to evaluate the potential of nanoniosomes as drug delivery carriers and optimize their formulation for specific therapeutic applications [50]. 

In 2022, Saleh et al. characterized *Lippia citriodora* essential oil-loaded niosomes with different molar ratios of surfactants (Span 60/Tween 60). It was found that the Span 60/Tween 60 ratio significantly affected the PDI, zeta potential, and particle size. In addition, they selected the niosome composed of Span 60 and cholesterol as the optimal formulation due to its high encapsulation efficiency (>85%) and its low nanometric size of <200 nm [28]. Pando et al. [51] showed a clear relationship between nanonoisome size and surfactant type. According to their reports, smaller head groups and longer alkyl chains of the surfactant lead to a larger vesicle size. Span 60 is a non-ionic surfactant with a long hydrophobic moiety and low water solubility, while Tween 60 is a surfactant with a hydrophilic head group. Although most of the reported formulations of nanoniosomes have shown that a higher amount of Tween 60 compared with Span 60 leads to a decrease in nanoniosome size, we observed the opposite result. It seems that the hydrophilic nature of GA increases the amount of binding to F3 (with a higher ratio of Tween 60) and hence increases both the nanoniosome size and the amount of encapsulation. Thus, as the amount of GA encapsulated increases, the hydrodynamic size of the particles also increases, which might be due to the diffusion of GA between the surfactant tails within the bilayer of the niosomes. Shatalebi et al. (2010) mentioned that the smaller size of the niosomes may provide a larger exposed surface area and result in a greater likelihood of drug leakage from vesicles and a lower encapsulation efficiency [52]. However, more detailed studies are needed to confirm this. 

SEM analysis revealed an average size of 65 nm for the GAN without aggregation. This size measurement obtained from SEM closely matched the size estimation obtained from the Zetasizer Nano, confirming that the size of formula F2 was below 100 nm by two independent techniques.

The FTIR results indicate that the GA molecules were effectively incorporated into the nanoniosomes in the F2 formulation. The disappearance of specific peaks of GA in the FTIR spectrum of GAN suggests successful drug entrapment within the niosomal formulation.

Notably, the data fitting of the GA release successfully matched the Korsmeyer–Peppas model. This finding highlights the promising nature of the F2 formulation in delivering GA in a controlled and sustained manner, which could be advantageous for targeted therapeutic effects and minimizing potential side effects.

According to the antimicrobial test, the new formulation enhanced the antimicrobial activity of GA, making it a promising candidate for combating bacterial infections caused by *Escherichia coli* and *Pseudomonas aeruginosa*. In a study by Zhang et al. (2019) GA liposomes showed antibacterial properties against *Escherichia coli*. These results highlight the importance of encapsulation in improving the efficiency of GA [15].

In this study, the findings demonstrated that the trapping of GA in niosomes (F2) increased the anticancer activity of GA against B16F10 cells. The dose-dependent inhibition of cell growth by the F2 formulation indicated its effectiveness in targeting and impacting cancer cells. Additionally, the PEG coating may enhance their binding to cancer cells, increase cellular absorption in the niosomes, and boost the release rate in these cells on the surface of the niosome vesicles. In 2013, Su et al. investigated the effects of GA on *B16F10* melanocyte cells. Their results indicated that GA was slightly cytotoxic to *B16F10* cells at a concentration higher than 200 μM [53], while it was nontoxic at lower than 100 μM. This finding is consistent with the results of this study. Also, Su et al. investigated the effects of GA on melanogenesis and its molecular mechanism. The results showed that GA inhibits tyrosinase activity by MITF and the downregulation of other melanogenesis-related proteins in *B16F10* cells [53]. Also, the findings indicate GA reduces dopaquinone (DQ) to L-DOPA through redox cycling, similar to ascorbic acid. Su et al. mentioned that GA also may serve as a substrate and gradually oxidize even in the absence of L-DOPA. Thus, it significantly increases the oxidation rate [8]. As mentioned, to overcome this limitation, the encapsulation of GA may protect it from oxidation. Chaikul et al. (2019) evaluated the anti-melanoma and anti-tyrosinase activities of GA loaded in neutral (Brij 52/cholesterol at 7:3) and cationic CTAB niosomes (Brij 52/cholesterol/cetyltrimethylammonium bromide at 7:3:0.65) compared with free GA. The results showed the highest melanin suppression effect (55.92 ± 4.92% of control) for the CTAB niosome by the inhibition of TYR (53.18 ± 3.67% of control) [54]. This study found another niosomal formulation with a lower size that enhances the anti-tyrosinase and anti-melanoma activity of GA. 

## 4. Materials and Methods

### 4.1. Materials

Gallic acid (purity ≥ 95%), sorbitan monostearate (Span^TM^ 60), polyoxyethylene sorbitan monolaurate (Tween^®^ 60), 2,2-diphenyl-1-picryl-hydrazine-hydrate (DPPH˙) reagent, 3-(4, 5-dimethyl-2-thiazolyl)-2, dimethyl sulfoxide (DMSO), 5-diphenyl-2H-tetrazolium bromide (MTT), phosphate-buffered saline tablet (PBS) (dissolved in deionized water), L-3,4-dihydroxyphenylalanine (DOPA), gentamycin, penicillin, and streptomycin were obtained from Sigma-Aldrich (St. Louis, MO, USA); cholesterol (purity, 95%), polyethylene glycol (PEG) 4000, and other chemicals were purchased from Merck (Darmstadt, Germany); B16F10 cell lines were provided by the Pasteur Institute Cell Bank (Tehran, Iran); fetal bovine serum, and Dulbecco’s Modified Eagle Medium (DMEM) were purchased from GIBCO (Billings, MT, USA); three strains of Gram-negative—*Klebsiella pneumoniae* (ATCC 10031), *Pseudomonas aeruginosa* (ATCC 27853), and *Escherichia coli* (ATCC 11333)—were obtained from the Iranian Biological Resource Center.

### 4.2. Preparation of GAN 

To prepare niosomal formulations, the thin film hydration method was used following the method of Ravalika et al. [55] with some modifications. Different formulations were prepared at a fixed ratio of cholesterol to surfactant (1:1) but with different surfactant types (Span^TM^ 60 and Tween^®^ 60) in the presence of 1% PEG. The molar ratios of cholesterol, Span^TM^ 60, and Tween^®^ 60 were as follows: F1 = 1:0.5:0.5, F2 = 1:1:0, and F3 = 1:0:1. Briefly, 15 mM surfactants (F1: 7.5 mM Span 60 (161.482 mg), 7.5 mM Tween 60 (491.625 mg); F2: 15 mM Span 60 (322.96 mg); F3: 15 mM Tween 60 (983.25 mg)), and 15 mM cholesterol (289.98 mg) along with 1.5 mM GA (12.75 mg) and 1% PEG (0.06 g) were dissolved in 40 mL of chloroform and 10 mL of methanol at 25 °C. Then, the chloroform was evaporated by a rotary evaporator (Heidolph Hei-VAP Advantage, Heidelberg, Germany) under reduced pressure at 60 °C and 150 rpm for 45 min. The resulting dried film was then hydrated by manual shaking in 50 mL of phosphate-buffered saline (10 mM, pH 7.2) for 30 min. All dispersions were sonicated by an ultrasonic homogenizer (Sonopuls HD-4200, Bandelin Co., Berlin, Germany) in four cycles on (180 s) and off (60 s), resulting in a unilamellar niosomal dispersion formation, and stored at 4 °C for further characterization. Blank nanoniosomal dispersions (F01, F02, and F03) were prepared by the same method but without the inclusion of GA. 

### 4.3. Characterization of GAN

#### 4.3.1. Dynamic Light Scattering (DLS) Measurement

The mean particle size, zeta potential, and polydispersity index (PDI) of the GAN formulations were assessed using DLS with a laser diffraction analyzer (SZ-100, Horiba, Osaka, Japan) at 633 nm and 25 °C.

#### 4.3.2. Encapsulation Efficiency Evaluation

The encapsulation efficiency (*EE*) of GAN formulations is calculated according to the following equation [56]:

*EE = (Total drug − untrapped drug)/Total drug ×* 100

After ultra-filtration (20 min at 4000× *g*) using Ultracel-30K Millipore filters, the absorbance of free GA in the supernatant was measured at 270 nm using UV–visible spectroscopy (Cary 50, Varian, Belrose, Australia), and the amount of GA was calculated on the basis of the standard curve. 

#### 4.3.3. Scanning Electron Microscopy

The niosomal dispersions were diluted with deionized water and placed on a silicon wafer. After placing them in the dryer overnight, a thin layer of gold was deposited on the dried samples and analyzed using a scanning electron microscope (Tescan Vega 3; Tescan Co., Brno, Czech Republic).

#### 4.3.4. Fourier-Transform Infrared (FTIR) Spectroscopy

The samples were scanned (4000 to 400 cm^−1^ with a resolution of 4 cm^−1^) at room temperature using an FTIR spectrophotometer (Tensor II, Bruker, Ettlingen, Germany) and the KBr disk method.

### 4.4. In Vitro Kinetics Study of GA Releasing

To investigate the in vitro release of GA from the optimal formulation F2, 5 mL of the nanoniosomal suspension was added to dialysis bags with a cut-off of 12 kDa. Then, they were immersed in 100 mL of PBS (pH of 7.4) and maintained at 37 °C. The aliquots were withdrawn at predetermined intervals and the PBS was replaced with fresh PBS. The absorbance of released GA was measured using a UV–Vis spectrophotometer at 270 nm. The amount of released drug was estimated using the standard curve, and the cumulative release percentage was plotted against time. Furthermore, the release kinetics were analyzed using Korsmeyer–Peppas, Higuchi, first-order, and zero-order models. To assess the mechanism of GA release from the niosomal dispersions, the data were fitted to the following models [57]:

1. Zero-order model: Q_t_ = k_0_ t,
2. First-order model: logQ_t_ = kt/2.303,
3. Korsmeyer–Peppas model: M_t_/M_∞_ = k t^n^,
4. Higuchi model: Q_t_ = k_H_ t^1/2^,
5. Hixson–Crowell model: W_o_^1/3^ − W_t_^1/3^ = k t

where Q_t_ is the quantity of GA dissolved at time t, M_t_/M_∞_ is the fraction of GA released at the time t, W_0_ is the initial amount of GA in the formulation, W_t_ is the remaining amount of GA in the formulation at time t, and k is a constant in all equations.

### 4.5. Antimicrobial Activity Evaluation

In this study, we investigated the antibacterial activity of GA and GAN against three strains of Gram-negative bacteria, namely *Klebsiella pneumoniae* (ATCC 10031), *Pseudomonas aeruginosa* (ATCC 27853), and *Escherichia coli* (ATCC 11333). The antibacterial assays were conducted using the serial dilutions of 1500 µM stock solution of niosomal dispersion and gentamycin (ranging from 23 to 750 µM) with 900 µL Mueller Hinton Broth media in 96-well plates. Three replicates of each dilution were prepared. Subsequently, 10 µL of bacterial suspension (108 CFU/mL) was added to each well and incubated at 37 °C for 24 h. Negative control wells contained 200 µL of culture media and 10 µL of bacterial suspension with blank niosomal F2 dispersion. The MIC of GA and GAN was defined as the lowest concentration of the compounds that inhibited bacterial growth, and the MBC was determined by sub-culturing the broth dilutions. 

### 4.6. Anti-melanoma Activity Measurement by MTT Assay

The *B16F10* melanoma cells were cultured in a growth medium containing antibiotics (1%) and FBS (10%) at 37 °C. For cell viability measurement by MTT assay [58,59,60,61,62,63], *B16F10* cells were seeded in 96-well plates at a density of 1 × 10^4^ cells/well and incubated at 37 °C in a 5% CO_2_ incubator for 24 h. Then, different concentrations (10, 20, 40, 60, 80, and 100 μM) of GA, the GAN formulation, and blank niosomal dispersions were added to the respective wells in triplicate and further incubated in a 5% CO_2_ incubator at 37 °C for 48 h. After incubation, 20 µL of MTT solution (5 mg/mL in PBS) was added and incubated for three hours in a 5% CO_2_ incubator at 37 °C. Then, after removing the medium, DMSO (200 µL) was added to each well and incubated for 5–10 min at 25 °C. Finally, the formazan formation was measured by an ELISA microplate reader (Biotek, Santa Clara, CA, USA) at 595 nm, and the cell viability was calculated using the following equation:

*Cell viability (%) = (Absorbance _treatment_ − Absorbance _blank_)/(Absorbance _control_ − Absorbance _blank_) ×* 100

### 4.7. Melanin Assay

The melanin content in melanoma cells was determined using a method described by Ullah et al. [64]. Briefly, *B16F10* cells (1 × 10^5^) were seeded and incubated for 24 h. Then, the cells were treated with GA and GAN at 40 µM and further incubated for 72 h. After removing the medium, the cells were washed with PBS. Then, the cell pellets were dissolved with DMSO (10%) containing NaOH (1 N) and kept for one hour in a UV sterilizer at 70 °C. Finally, the melanin content was measured using an ELISA reader at 405 nm. 

### 4.8. Intracellular TYR Activity 

Intracellular TYR activity assay was measured using a described method by Eghbali et al. [65]. Briefly, the cells (1 × 10^5^) were seeded, incubated for 24 h, and treated with GA and GAN at 40 µM. Then, after detaching by trypsin, the cells were washed with PBS and lysed with PBS containing Triton X-100 (1%). The lysates were centrifuged for 20 min at 10,000 rpm at 4 °C, and 100 μL of each lysate was mixed with 30 μL of 5 mM DOPA in 96-well plates and incubated for two hours at 37 °C. Finally, the oxidation of DOPA to DOPA chrome, an indicator of TYR activity, was analyzed by an ELISA Reader (BMG Labtech, Ortenberg, Germany) at 475 nm.

### 4.9. Statistical Analysis

All experiments were performed in triplicate, and results are reported as means ± standard deviation. Statistical analysis was conducted by SPSS version 19.0 using one-way analysis of variance (ANOVA) followed by Duncan’s multiple range tests (*p* < 0.05). Also, to investigate the relationship between size, EE%, and zeta potential, Pearson correlation coefficients were calculated.

## 5. Conclusions

Given that GA is widely used in pharmacy, cosmetics, the food industry, and medical and chemical research, it is necessary to identify formulations that increase the stability and effectiveness of GA. Because of the advantages of niosomes in skincare products, this study aimed to find a small-sized drug delivery system for GA that could penetrate the skin and be used in cosmetic products. Also, this investigation aimed to provide an appropriate nano platform for enhancing anti-melanoma, anti-tyrosinase, and antibacterial GA. The results suggest that the Span 60/cholesterol niosomal formulation is a suitable GA delivery system for skin problems with potent in vitro antibacterial, anti-melanoma, and anti-tyrosinase activities. This new formulation (GAN–Span 60), with a small size of 80 nm, good stability, an acceptable EE%, and suitable drug release, can be considered as a potential drug delivery system in further development studies of new dermatological products addressing skin problems.

## Figures and Tables

**Table 1:** Characteristics of three niosomal formulations.

Formulation	Cholesterol/ Surfactant (Molar Ratio)	Drug (Mm)	Cholesterol: Span 60 to Tween 60	Zeta Potential (mv) ± SD	Size ± SD	PDI ± SD	EE% ± SD
F1	1-1	1.5	1:0.5:0.5	−24.96 ± 2.1	169.8 ± 4.1	0.22 ± 0.1	80 ± 4
F2	1-1	1.5	1:1:0	−44.78 ± 2.3	80.2 ± 5.7	0.26 ± 0.01	75 ± 3
F3	1-1	1.5	1:0:1	−9.78 ± 1.1	276.8 ± 7.4	0.34 ± 0.04	96 ± 6
F01 (blank)	1-1	0	1:0.5:0.5	−3.4 ± 1.5	118.3 ± 4.3	0.23 ± 0.08	---
F02 (blank)	1-1	0	1:1:0	−31.2 ± 3.6	64.2 ± 3.1	0.21 ± 0.02	---
F03 (blank)	1-1	0	1:0:1	−8.65 ± 2.0	180 ± 1.2	0.3 ± 0.01	---

**Table 2:** Kinetic data of in vitro release from F2 formulation to find the best-fitted model.

Mathematical Model for Releasing	First Stage (Up to 60% Release)		
	R^2^	k	n
Korsmeyer–Peppas	0.98	0.025	0.63
Higuchi	0.94	15.6	
Hixson–Crowell	0.85	0.06	
First-order	0.96	0.02	
Zero-order	0.91	2.7	

**Table 3:** Results of the microbiological test. (MCB and MIC represent the average of three samples).

Test Groups	MBC (µM)			MIC (µM)		
	*E. coli*	*P. aeruginosa*	*K. pneumonia*	*E. coli*	*P. aeruginosa*	*K. pneumonia*
GA	750	750	>750	375	375	750
GAN (F2 formulation)	46	375	750	23	187	750
Gentamycin	131	524	262	131	262	131

## Data Availability

Data is contained within the article.
